# Exploratory composite endpoint demonstrates benefit of trilaciclib across multiple clinically meaningful components of myeloprotection in patients with small cell lung cancer

**DOI:** 10.1002/ijc.33705

**Published:** 2021-07-10

**Authors:** Manuel Dómine Gómez, Tibor Csőszi, Jana Jaal, Iveta Kudaba, Krasimir Nikolov, Davorin Radosavljevic, Jie Xiao, Janet K. Horton, Rajesh K. Malik, Janakiraman Subramanian

**Affiliations:** ^1^ Medical Oncology Department Hospital Universitario Fundación Jimenez Diaz Madrid Spain; ^2^ County Oncology Centre Hetenyi Geza Korhaz Szolnok Hungary; ^3^ Institute of Clinical Medicine, Department of Hematology and Oncology University of Tartu Tartu Estonia; ^4^ Chemotherapy and Haematology Clinic Riga East Clinical University‐Latvian Oncology Center Riga Latvia; ^5^ Department of Medical Oncology Complex Oncology Center Burgas Bulgaria; ^6^ Clinic for Medical Oncology Institute for Oncology and Radiology of Serbia Belgrade Serbia; ^7^ Clinical Development G1 Therapeutics, Inc. Research Triangle Park North Carolina USA; ^8^ Division of Oncology, Saint Luke's Cancer Institute University of Missouri Kansas City Missouri USA

**Keywords:** chemotherapy, myelopreservation, myeloprotection, myelosuppression, small cell lung cancer, trilaciclib

## Abstract

Chemotherapy‐induced myelosuppression is an acute, dose‐limiting toxicity of chemotherapy regimens used in the treatment of extensive‐stage small cell lung cancer (ES‐SCLC). Trilaciclib protects haematopoietic stem and progenitor cells from chemotherapy‐induced damage (myeloprotection). To assess the totality of the myeloprotective benefits of trilaciclib, including analysis of several clinically relevant but low‐frequency events, an exploratory composite endpoint comprising five major adverse haematological events (MAHE) was prospectively defined: all‐cause hospitalisations, all‐cause chemotherapy dose reductions, febrile neutropenia (FN), prolonged severe neutropenia (SN) and red blood cell (RBC) transfusions on/after Week 5. MAHE and its individual components were assessed in three randomised, double‐blind, placebo‐controlled Phase 2 trials in patients receiving a platinum/etoposide or topotecan‐containing chemotherapy regimen for ES‐SCLC and in data pooled from the three trials. A total of 242 patients were randomised across the three trials (trilaciclib, n = 123; placebo, n = 119). In the individual trials and the pooled analysis, administering trilaciclib prior to chemotherapy resulted in a statistically significant reduction in the cumulative incidence of MAHE compared to placebo. In the pooled analysis, the cumulative incidences of all‐cause chemotherapy dose reductions, FN, prolonged SN and RBC transfusions on/after Week 5 were significantly reduced with trilaciclib vs placebo; however, no significant difference was observed in rates of all‐cause hospitalisations. Additionally, compared to placebo, trilaciclib significantly extended the amount of time patients remained free of MAHE. These data support the myeloprotective benefits of trilaciclib and its ability to improve the overall safety profile of myelosuppressive chemotherapy regimens used to treat patients with ES‐SCLC.

**Abbreviations**
aRR: adjusted rate ratio
CDK4/6: cyclin‐dependent kinase 4 and 6
CI: confidence interval
CIM: chemotherapy‐induced myelosuppression
ECOG PS: Eastern Cooperative Oncology Group performance status
ESA: erythropoiesis‐stimulating agent
ES‐SCLC: extensive‐stage small cell lung cancer
FN: febrile neutropenia
G‐CSF: granulocyte colony‐stimulating factor
HR: hazard ratio
HSPC: haematopoietic stem and progenitor cell
IV: intravenous
MAHE: major adverse haematological event
QD: once daily
RBC: red blood cell
SCLC: small cell lung cancer
SN: severe neutropenia

**What's new?**

Chemotherapy‐induced myelosuppression is common in patients with small cell lung cancer and both the ensuing chemotherapy dose reductions/delays and the supportive care interventions are associated with risks. Trilaciclib has been found to decrease the incidence of chemotherapy‐induced myelosuppression in three randomised, placebo‐controlled clinical trials of patients with extensive‐stage small cell lung cancer. Here, to further assess the myeloprotective effects of trilaciclib, an exploratory composite endpoint of major adverse haematological events was developed. Compared with placebo, trilaciclib reduced the cumulative incidence of major adverse haematological events and increased time to first event, indicating that trilaciclib improves the overall safety of myelosuppressive chemotherapy regimens.

## 1 INTRODUCTION

Small cell lung cancer (SCLC) is an aggressive disease, with approximately 60% to 70% of patients having extensive‐stage SCLC (ES‐SCLC) at diagnosis.^1^ Although platinum‐based chemotherapy regimens have been the cornerstone of treatment for patients with ES‐SCLC,^1^, ^2^ the addition of immunotherapy to chemotherapy has led to significant improvements in clinical outcomes and represents a new standard of care for patients with otherwise limited therapeutic options.^3^, ^4^, ^5^ Most chemotherapeutic agents exert their effects by targeting highly proliferative cells; therefore, in addition to tumour cells, high‐turnover normal tissues, such as hair follicles, mucosa and bone marrow, are also damaged. Chemotherapy‐induced damage of haematopoietic stem and progenitor cells (HSPCs) in the bone marrow leads to myelosuppression, a common and sometimes life‐threatening complication that most commonly manifests as neutropenia, anaemia and/or thrombocytopenia.^6^ Indeed, haematological adverse events are among the most frequently reported toxicities associated with chemotherapy‐based SCLC treatment regimens.^7^ Chemotherapy‐induced myelosuppression (CIM) can increase the risk of serious infections and bleeding and negatively impact patients' quality of life.^8^, ^9^, ^10^, ^11^

CIM is usually managed with chemotherapy dose reductions and/or delays, which may reduce the dose intensity of chemotherapy and potentially impair its therapeutic efficacy.^12^, ^13^, ^14^ Moreover, supportive care interventions for CIM are generally used after the onset of symptoms and only address the deficiency of a single blood cell lineage. Such interventions also carry their own set of risks and limitations. For example, the use of erythropoiesis‐stimulating agents (ESAs) to manage chemotherapy‐induced anaemia is only effective in approximately 60% of patients and is associated with an increased risk of thromboembolic events.^15^, ^16^ Red blood cell (RBC) and platelet transfusions, used to treat anaemia and thrombocytopenia, respectively, are burdensome to patients and carry risks of transfusion reactions, thromboses, alloimmunisation and immunosuppression, and infection.^9^, ^17^, ^18^, ^19^ Although primary prophylaxis with granulocyte colony‐stimulating factors (G‐CSFs) can reduce the risk of febrile neutropenia (FN) and improve overall survival, use of G‐CSFs can lead to adverse effects such as bone pain.^20^, ^21^, ^22^ In addition to these challenges, the use of supportive care interventions and the need for hospitalisations owing to CIM and its consequences can incur substantial healthcare resource use and financial costs, resulting in a considerable economic burden on patients, caregivers and healthcare systems.^10^, ^23^, ^24^ A treatment that can proactively protect against CIM, thereby reducing the need for supportive care and hospitalisations, would therefore be particularly valuable.

Trilaciclib is an intravenous (IV) cyclin‐dependent kinase 4 and 6 (CDK4/6) inhibitor indicated to decrease the incidence of CIM. Trilaciclib transiently arrests CDK4/6‐dependent HSPCs in the G1 phase of the cell cycle during chemotherapy exposure, thereby protecting bone marrow function from chemotherapy‐induced damage (myeloprotection or myelopreservation).^25^, ^26^ The myeloprotective effects of trilaciclib differ from the myelosuppressive effects of oral CDK4/6 inhibitors approved for the treatment of hormone receptor–positive breast cancer. The chronic administration of oral CDK4/6 inhibitors results in myelosuppression owing to the sustained blockade of HSPC proliferation in the bone marrow^27^; by contrast, IV infusions of trilaciclib are completed within 4 hours prior to chemotherapy on each day chemotherapy is administered, allowing for more control over HSPC cycle arrest. As a result, HSPCs are protected from damage during exposure to chemotherapy, but are able to resume proliferation afterwards.^26^, ^28^ Trilaciclib has been evaluated in three independent, global, multicentre, randomised, double‐blind, placebo‐controlled Phase 2 trials in which patients with ES‐SCLC received trilaciclib or placebo prior to chemotherapy.^29^, ^30^, ^31^ In each individual trial, and in a pooled analysis of data from all three trials, administering trilaciclib prior to chemotherapy led to a reduction in CIM across multiple lineages, along with a reduction in the use of supportive care measures, and improvements in quality of life.^29^, ^30^, ^31^, ^32^ Notably, the observed myeloprotective effects of trilaciclib were observed in the absence of any detrimental effects on antitumour efficacy.^29^, ^30^, ^31^, ^32^

Because trilaciclib affects multiple haematological and associated outcomes, a predefined, exploratory composite endpoint was used to assess the totality of benefit with trilaciclib across several clinically meaningful components of myeloprotection. Here, we describe the development of this composite endpoint of major adverse haematological events (MAHE) and present results from each of the three SCLC trials, along with results from the pooled analysis.

## 2 PATIENTS AND METHODS

### 2.1 Study design and participants

The three randomised Phase 2 trials were conducted at multiple sites across the United States and Europe. Full details of the study designs have been reported previously.^29^, ^30^, ^31^ In study G1T28‐05 (NCT03041311), patients with newly diagnosed ES‐SCLC received IV trilaciclib 240 mg/m^2^ or placebo QD within 4 hours prior to chemotherapy (IV etoposide 100 mg/m^2^ on Days 1, 2 and 3, carboplatin area under the curve 5 and IV atezolizumab 1200 mg on Day 1) on Days 1‐3 for up to four 21‐day cycles, followed by maintenance therapy without trilaciclib or placebo (IV atezolizumab 1200 mg monotherapy on Day 1 of 21‐day cycles). In study G1T28‐02 (NCT02499770), patients with newly diagnosed ES‐SCLC received IV trilaciclib 240 mg/m^2^ or placebo once daily (QD) prior to chemotherapy administration (IV etoposide 100 mg/m^2^ on Days 1, 2 and 3, and carboplatin area under the curve 5 on Day 1) on Days 1‐3 of each 21‐day cycle. In study G1T28‐03 (NCT02514447), patients with previously treated ES‐SCLC received IV trilaciclib 240 mg/m^2^ or placebo QD prior to chemotherapy (0.75 or 1.5 mg/m^2^ topotecan) on Days 1‐5 of each 21‐day cycle.

In each study, eligible patients were aged ≥18 years with confirmed ES‐SCLC and had measurable disease by Response Evaluation Criteria in Solid Tumors version 1.1, adequate organ function and an Eastern Cooperative Oncology Group performance status (ECOG PS) of 0‐2. Patients were ineligible if they presented with symptomatic brain metastases requiring immediate treatment, and, for studies G1T28‐05 and G1T28‐02, if they had received prior systemic therapy for SCLC. For study G1T28‐03, patients were excluded if they had a history of topotecan treatment, and they must have had disease progression during or after first‐ or second‐line chemotherapy and been eligible to receive topotecan.

In each study, primary prophylaxis with G‐CSF and use of ESAs was prohibited in cycle 1, although therapeutic G‐CSF was allowed; after cycle 1, supportive care, including G‐CSF and ESAs, was allowed as needed. RBC and platelet transfusions were allowed per investigator discretion throughout the entire treatment period.

Each study was conducted in accordance with the Declaration of Helsinki and Good Clinical Practice guidelines of the International Council for Harmonisation, and the protocols and all study‐related materials were approved by the institutional review board or independent ethics committee of each participating site. All patients provided written informed consent.

### 2.2 MAHE composite endpoint

The MAHE composite endpoint is comprised of five individual components that are clinically relevant but low‐frequency consequences of CIM (Table 1). These include all‐cause hospitalisations, all‐cause chemotherapy dose reductions, FN, prolonged severe neutropenia (SN; defined as absolute neutrophil count <0.5 × 10^9^ cells/L lasting >5 days) and RBC transfusions on/after Week 5. RBC transfusions before Week 5 were excluded to ensure that analyses of potential benefit were not confounded by the residual effect of previous treatment.

**TABLE 1:** Components of the MAHE composite endpoint

All‐cause hospitalisations (per week)^a^
All‐cause chemotherapy dose reductions (per cycle)^b^
Febrile neutropenia (per week)^a^
Prolonged severe neutropenia (ANC <0.5 × 10^9^ cells/L lasting >5 days; per cycle)^b^
RBC transfusions on/after Week 5 (per week)^b^

Abbreviations: ANC, absolute neutrophil count; MAHE, major adverse haematological event; RBC, red blood cell.

^a^ Captured in assessment of safety.

^b^ Myelosuppression endpoints.

For each MAHE component, the number of events was counted as the number of events (all‐cause hospitalisations, FN, RBC transfusions on/after Week 5) or cycles (all‐cause chemotherapy dose reductions, prolonged SN) with a unique start date during the treatment period. The cumulative incidence of MAHE was obtained by summing the total number of events across the prespecified components. The cumulative incidence of MAHE and its individual components was assessed for each individual trial and using pooled data across the three trials, including a subgroup analysis conducted according to age (<65 years, ≥65 years), sex, ethnic group (White, non‐White) and region (USA, non‐USA). The time to first occurrence of a MAHE, defined as the time from the date of randomisation to the first event observed among all MAHE components, was assessed for each individual trial and using pooled data.

### 2.3 Statistical analysis

The aim of the pooled analysis was to understand the effects of trilaciclib with greater statistical precision, particularly for low‐frequency endpoints such as those related to RBCs and for specific patient populations with limited numbers in the individual trials. The pooled analysis set included all randomised patients in study G1T28‐05, all randomised patients in the Phase 2 part of study G1T28‐02 and patients receiving trilaciclib or placebo plus topotecan 1.5 mg/m^2^ in the Phase 2 part of study G1T28‐03. Breslow‐Day testing was used to assess the statistical validity of data integration.^32^

The treatment effect of trilaciclib compared to placebo was assessed using a negative binomial regression model, adjusting for duration of treatment in weeks or number of cycles. To adjust for potential variability between patients, the models included common stratification factors of ECOG PS (0 or 1 vs 2 in the pooled analysis and individual studies), presence of brain metastases (yes vs no in the pooled analysis and G1T28‐05) and sensitivity to first‐line treatment (sensitive vs resistant in G1T28‐03) as these stratification factors have previously been shown in SCLC trials to predict treatment outcomes.^33^, ^34^, ^35^ Study (G1T28‐05, G1T28‐02 and G1T28‐03 in the pooled analysis) was also included as a fixed effect in order to adjust for potential variability between studies. The analyses of cumulative incidence of FN, prolonged SN and RBC transfusions included corresponding baseline laboratory values as covariates. Adjusted rate ratios (aRRs) for trilaciclib vs placebo and 95% confidence intervals (CIs) were generated, along with 2‐sided *P*‐values. For subgroup analyses, treatment‐by‐subgroup interactions were tested using a negative binomial method adjusting for duration of treatment in weeks, with stratification factors (ECOG PS [0 or 1 vs 2], presence of brain metastases [yes vs no] and study [G1T28‐02, G1T28‐03 and G1T28‐05]), treatment, subgroup and treatment‐by‐subgroup interaction as fixed effects. Time to first MAHE was assessed using Kaplan‐Meier methodology, and descriptive statistics (hazard ratio [HR], 95% CI and 2‐sided *P*‐value) were generated.

## 3 RESULTS

### 3.1 Study participants

Between 12 October 2016 and 1 June 2018, 242 patients were randomised across the three studies (trilaciclib prior to chemotherapy, n = 123; placebo prior to chemotherapy, n = 119). Three patients were enrolled in both G1T28‐02 and G1T28‐03; however, only data from G1T28‐02 were included in the pooled analysis for these patients.

Patient demographics and baseline disease characteristics were generally comparable across the trilaciclib and placebo groups, with both populations having a median age of 64 years. Of the patients receiving trilaciclib/placebo, 98%/92% were White, 43%/48% were from the United States and 88%/90% had an ECOG PS of 0 or 1. There were slightly more men and current smokers in the trilaciclib group than in the placebo group (72% vs 61% and 40% vs 30%, respectively).^32^

The Breslow‐Day test result for homogeneity of the odds ratios for neutrophils (occurrence of SN) was *P* = .1049, and results for homogeneity of the odds ratios for RBC endpoints were *P* = .9241 (occurrence of Grade 3 or 4 decreased haemoglobin levels) and *P* = .3547 (occurrence of RBC transfusions on/after Week 5), suggesting that the treatment effect of trilaciclib on neutrophil‐ and RBC‐related endpoints was statistically consistent across the three trials.^32^

### 3.2 Cumulative incidence of MAHE

**FIGURE 1:**
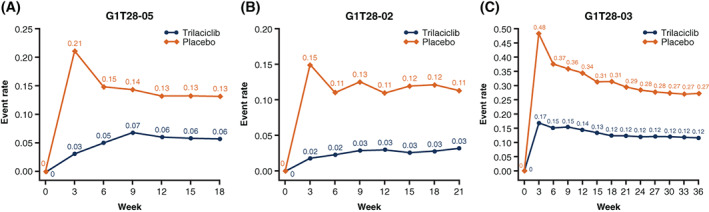
Cumulative incidence of MAHE by week in the individual studies (A‐C). MAHE, major adverse haematological event [Color figure can be viewed at wileyonlinelibrary.com]

In each of the three individual trials, aRRs of <1 indicated that, compared to placebo, administration of trilaciclib prior to chemotherapy resulted in a statistically significant reduction in the cumulative incidence of MAHE (Table 2). Cumulative incidence of MAHE was also clinically and statistically significantly lower for trilaciclib than for placebo in the pooled analysis (Table 2). Across all three individual trials the cumulative incidence of MAHE was higher in the placebo arm than in the trilaciclib arm by Week 3 and remained higher throughout the treatment period (up to Week 18 in study G1T28‐05, Week 21 in G1T28‐02 and Week 36 in G1T28‐03; Figure 1).

**TABLE 2:** Cumulative incidence of MAHE composite endpoint

Study	MAHE composite endpoint (event rate per week^a^)		Adjusted rate ratio (95% CI)^b^	*P*‐value
	Trilaciclib	Placebo		
G1T28‐05	0.058 n = 54	0.132 n = 53	0.437 (0.253, 0.754)^c^	.0029
G1T28‐02	0.031 n = 39	0.106 n = 38	0.246 (0.118, 0.510)^d^	.0002
G1T28‐03	0.114 n = 32	0.267 n = 29	0.455 (0.234, 0.884)^e^	.0201
Pooled	0.054 n = 123	0.139 n = 119	0.355 (0.245, 0.513)^f^	<.0001

Abbreviations: CI, confidence interval; ECOG PS, Eastern Cooperative Oncology Group performance status; MAHE, major adverse haematological event; n, number of patients.

^a^ Data are event rates per week, calculated as the total number of events divided by the total duration in weeks.

^b^ Calculated using the negative binomial method, adjusting for duration of treatment in weeks.

^c^ Stratification factors of ECOG PS (0 or 1 vs 2) and presence of brain metastases (yes vs no) included as fixed effects.

^d^ Stratification factor of ECOG PS (0 or 1 vs 2) included as a fixed effect.

^e^ Stratification factors of ECOG PS (0 or 1 vs 2) and sensitivity to first‐line treatment included as fixed effects.

^f^ Stratification factors of ECOG PS (0 or 1 vs 2), presence of brain metastases (yes vs no) and study (G1T28‐05, G1T28‐02, G1T28‐03) included as fixed effects.

Subgroup analysis of the MAHE composite endpoint in the pooled dataset indicated that, compared to placebo, administering trilaciclib consistently reduced the cumulative incidence of MAHE across all subgroups evaluated (Table 3). None of the treatment‐by‐subgroup interactions were statistically significant (all *P* > .05), indicating that trilaciclib had similar effects vs placebo regardless of baseline characteristics of age, sex, ethnic group and region.

**TABLE 3:** Pooled analysis of cumulative incidence of MAHE by subgroup

	Trilaciclib	Placebo
Age, n		
<65 years	66	61
Event rate^a^	0.051	0.102
Adjusted rate ratio (95% CI)^b^	0.461 (0.265, 0.801)	
≥65 years	57	58
Event rate^a^	0.058	0.176
Adjusted rate ratio (95% CI)^b^	0.303 (0.185, 0.496)	
Sex, n		
Male	89	73
Event rate^a^	0.041	0.102
Adjusted rate ratio (95% CI)^b^	0.330 (0.196, 0.555)	
Female	34	46
Event rate^a^	0.085	0.204
Adjusted rate ratio (95% CI)^b^	0.466 (0.286, 0.760)	
Ethnic group, n		
White	120	110
Event rate^a^	0.054	0.140
Adjusted rate ratio (95% CI)^b^	0.346 (0.236, 0.506)	
Non‐White	3	9
Event rate^a^	0.082	0.117
Adjusted rate ratio (95% CI)^b^	0.678 (0.105, 4.363)	
Region		
USA, n	53	57
Event rate^a^	0.074	0.179
Adjusted rate ratio (95% CI)^b^	0.401 (0.251, 0.639)	
Non‐USA, n	70	62
Event rate^a^	0.039	0.102
Adjusted rate ratio (95% CI)^b^	0.328 (0.185, 0.581)	

Abbreviations: CI, confidence interval; ECOG PS, Eastern Cooperative Oncology Group performance status; MAHE, major adverse haematological event.

^a^ Data are event rates per week, calculated as the total number of events divided by the total duration in weeks.

^b^ Calculated using the negative binomial method, adjusting for duration of treatment in weeks, with stratification factors of ECOG PS (0 or 1 vs 2), presence of brain metastases (yes vs no) and study (G1T28‐05, G1T28‐02, G1T28‐03) included as fixed effects.

### 3.3 Cumulative incidence of individual MAHE components

The cumulative incidences of the separate MAHE components in the individual studies and in the pooled analysis are shown in Table 4. Event rates of all‐cause chemotherapy dose reductions were significantly reduced for trilaciclib vs placebo in the pooled analysis and in studies G1T28‐05 and G1T28‐02. Event rates of prolonged SN were significantly reduced for trilaciclib vs placebo in the pooled analysis and in studies G1T28‐05 and G1T28‐03. Event rates for RBC transfusions on/after Week 5 were significantly reduced for trilaciclib vs placebo in the pooled analysis and in studies G1T28‐02 and G1T28‐03. The event rate of FN for trilaciclib vs placebo was significantly reduced in the pooled analysis and was numerically reduced in the individual trials. There was no significant difference between the trilaciclib and placebo groups in the rates of all‐cause hospitalisations in the pooled analysis or in the individual trials.

**TABLE 4:** Cumulative incidence of individual MAHE components

Study		n	All‐cause hospitalisations (per week)	All‐cause chemo dose reductions (per cycle)	FN (per week)	Prolonged SN (per cycle)	RBC transfusions on/after Week 5 (per week)
G1T28‐05	Event rate^a^						
	Trilaciclib	54	0.032	0.021	0.002	0.005	0.017
	Placebo	53	0.030	0.085	0.004	0.170	0.026
	Adjusted rate ratio (95% CI)^b^ ^,^ ^c^		1.087 (0.472, 2.507)	0.242 (0.079, 0.742)	0.405 (0.042, 3.929)	0.032 (0.004, 0.237)	NE
	*P*‐value		.8443	.0130	.4357	.0007	.1954
G1T28‐02	Event rate^a^						
	Trilaciclib	39	0.018	0.022	0.002	0.000	0.005
	Placebo	38	0.022	0.084	0.007	0.142	0.019
	Adjusted rate ratio (95% CI)^b^ ^,^ ^d^		0.611 (0.179, 2.087)	0.250 (0.079, 0.796)	0.052 (0.000, 8.901)	NE	0.190 (0.047, 0.777)
	*P*‐value		.4318	.0189	.2597	.9998	.0208
G1T28‐03	Event rate^a^						
	Trilaciclib	32	0.020	0.051	0.004	0.081	0.026
	Placebo	29	0.035	0.116	0.016	0.223	0.063
	Adjusted rate ratio (95% CI)^b^ ^,^ ^e^		0.536 (0.170, 1.687)	0.414 (0.146, 1.168)	0.225 (0.033, 1.528)	0.366 (0.168, 0.800)	0.441 (0.210, 0.924)
	*P*‐value		.2863	.0956	.1270	.0118	.0302
Pooled	Event rate^a^						
	Trilaciclib	123	0.024	0.028	0.002	0.020	0.015
	Placebo	119	0.028	0.093	0.008	0.171	0.031
	Adjusted rate ratio (95% CI)^b^ ^,^ ^f^		0.786 (0.427, 1.448)	0.263 (0.136, 0.507)	0.278 (0.078, 0.991)	0.097 (0.047, 0.202)	0.411 (0.230, 0.734)
	*P*‐value		.4403	<.0001	.0485	<.0001	.0027

Abbreviations: CI, confidence interval; ECOG PS, Eastern Cooperative Oncology Group performance status; FN, febrile neutropenia; MAHE, major adverse haematological event; NE, not evaluable; RBC, red blood cell; SN, severe neutropenia.

^a^ Calculated as the total number of events divided by the total duration in weeks, or the total number of cycles with an event divided by the total number of cycles.

^b^ Calculated using the negative binomial method, adjusting for duration of treatment in weeks or number of cycles.

^c^ Stratification factors of ECOG PS (0 or 1 vs 2) and presence of brain metastases (yes vs no) included as fixed effects.

^d^ Stratification factor of ECOG PS (0 or 1 vs 2) included as a fixed effect.

^e^ Stratification factors of ECOG PS (0 or 1 vs 2) and sensitivity to first‐line treatment included as fixed effects.

^f^ Stratification factors of ECOG PS (0 or 1 vs 2), presence of brain metastases (yes vs no) and study (G1T28‐05, G1T28‐02, G1T28‐03) included as fixed effects.

### 3.4 Time to first MAHE

**FIGURE 2:**
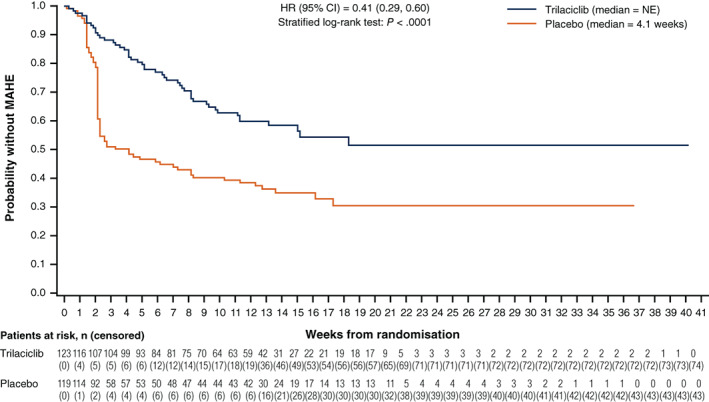
Time to first MAHE in the pooled analysis. CI, confidence interval; HR, hazard ratio; MAHE, major adverse haematological event; NE, not estimable [Color figure can be viewed at wileyonlinelibrary.com]

Administration of trilaciclib prior to chemotherapy significantly extended the amount of time patients remained free of MAHE compared to placebo. Median time to first MAHE was statistically significantly longer for trilaciclib vs placebo in the pooled analysis (not estimable vs 4.1 weeks; HR 0.41 [95% CI: 0.29, 0.60]; *P* < .0001; Figure 2).

## 4 DISCUSSION

CIM is common in patients with SCLC and can cause severe complications that negatively impact on patients' health and quality of life. Current management of CIM is often reactive in nature, occurring after the onset of myelosuppression symptoms and is associated with additional risks arising both from supportive care interventions and from chemotherapy dose reductions/delays.^9^, ^13^, ^16^, ^17^, ^18^, ^19^, ^22^

To better quantify the overall myeloprotective benefits of trilaciclib, a composite endpoint comprising five MAHE that are clinically relevant consequences of CIM was prospectively defined and assessed in three individual studies in patients with ES‐SCLC and in a pooled analysis. The clinically and statistically significant reductions in the MAHE composite endpoint and its individual components support the utility of trilaciclib in protecting HSPCs from chemotherapy‐induced damage, as reflected by significant reductions in rates of all‐cause chemotherapy dose reductions, FN, prolonged SN and RBC transfusions on/after Week 5. Together with previously reported data, these findings support the myeloprotective benefits of trilaciclib in patients with ES‐SCLC undergoing chemotherapy.

Although the cumulative incidence of MAHE was significantly reduced with the addition of trilaciclib prior to chemotherapy, trilaciclib did not have a statistically significant effect on the rate of all‐cause hospitalisations compared to placebo in the individual studies or the pooled analysis. This could be because this endpoint included all hospitalisations, including those due to reasons other than myelosuppression and is therefore not sensitive enough to register changes resulting from the administration of trilaciclib. Indeed, a separate ad hoc analysis of pooled safety data from these three SCLC trials showed that significantly fewer patients receiving trilaciclib were hospitalised specifically owing to CIM or sepsis compared to those receiving placebo, in line with the myeloprotective mechanism of action of trilaciclib.^32^ Hospital admissions and readmissions are common in oncology care and are often associated with myelosuppression symptoms.^36^, ^37^ The need to better identify and reduce all preventable hospitalisations due to myelosuppression is therefore a key target area for oncology practices. Notably, CIM and its complications are more likely to occur in older patients; as a result, these patients are more often hospitalised because of life‐threatening infectious complications.^38^, ^39^ Subgroup analyses have shown that the myeloprotective benefits of trilaciclib vs placebo are observed irrespective of age (<65 or ≥65 years), with greater effects observed among patients aged ≥65 years.^40^ Indeed, in this analysis, the cumulative incidence of MAHE was approximately three times lower among patients aged ≥65 years who received trilaciclib compared to older patients who received placebo, suggesting trilaciclib has the potential to minimise CIM and its complications, including hospitalisations, in older patients treated with myelosuppressive chemotherapy. Overall, hospitalisation remains an important component of the MAHE composite endpoint but may be better assessed by focussing on CIM/sepsis hospitalisations, both for the assessment of the myeloprotective effects of trilaciclib and, potentially, as a general tool to identify and address potentially avoidable hospitalisations due to myelosuppression.

Combined with previously reported findings of reduced CIM across multiple endpoints, reduced use of supportive care and improved quality of life with the addition of trilaciclib,^29^, ^30^, ^31^ the findings of the current analysis support the value of the MAHE composite endpoint in evaluating the efficacy of trilaciclib with regard to myeloprotection. Moreover, we suggest that this endpoint could also be evaluated and validated for use in other clinical situations as a metric to monitor the incidence and impact of multilineage myelosuppression. As the components of the MAHE composite endpoint are strongly associated with cytopenias that may compromise treatment outcomes and add to the burden of cancer on healthcare systems, the MAHE endpoint has the potential to be a clinically and economically important endpoint for evaluating oncology treatments in real‐world practice.

Regarding the individual MAHE constituents, FN is a rare but serious complication of chemotherapy that constitutes a major cause of morbidity, healthcare resource use and mortality. Even in the absence of fever, prolonged SN places patients at a high risk of serious infection,^8^, ^41^, ^42^ thus supporting the inclusion of prolonged SN and FN as components of the MAHE composite.

Monitoring the use of RBC transfusions as part of the MAHE endpoint is also informative. RBC transfusions are most frequently prescribed to patients receiving myelosuppressive chemotherapy owing to the presence of anaemia,^43^ with their continued use in patients with chemotherapy‐induced anaemia often linked to restrictive ESA labels and concerns that ESAs may increase the risk of tumour progression and/or mortality.^16^ However, RBC transfusions incur substantial economic costs and are burdensome to patients.^18^, ^44^ Blood supplies for transfusions are also finite, and sufficient availability is not always guaranteed, as recently illustrated by shortages during the COVID‐19 pandemic.^45^

Patients treated in real‐world practice commonly experience chemotherapy dose delays and reductions, for which cytopenias and their consequences are often the main cause.^46^ In this regard, monitoring dose reductions as part of the MAHE endpoint could provide a reliable index for ensuring standard‐of‐care dosing is maintained, alongside improved patient tolerability.

However, the concurrent use of supportive care measures must also be considered. It is important to note that in each of the three individual studies, cumulative rates of MAHE events decreased in the placebo groups after the first chemotherapy cycle. This most likely reflects the fact that supportive measures, including prophylactic GCSF (therapeutic GCSF was allowed in any cycle) and ESAs, were allowed after this time, as per protocol. Moreover, after the first cycle, the probability of remaining free from MAHE appeared to decline less steeply in the placebo group than in the trilaciclib group. This may be due to patients in the placebo group receiving more supportive care measures than those in the trilaciclib group. Indeed, a recent pooled analysis of the three studies showed that, across the treatment period, the use of G‐CSFs, ESAs and RBC transfusions on/after Week 5 was significantly higher among patients receiving placebo than those receiving trilaciclib (56.3% vs 28.5%, *P* < .0001; 11.8% vs 3.3%, *P* = .0252; 26.1% vs 14.6%, *P* = .0254, respectively).^47^

In addition to the impact of myelosuppression on patients' health and quality of life, the economic impact of haematological AEs and their management is also an important consideration for healthcare systems. It is notable, therefore, that a recent budget impact assessment for trilaciclib in decreasing the incidence of CIM in patients with ES‐SCLC found that the incremental cost of trilaciclib was projected to be largely offset by a reduction in the costs of managing AEs related to myelosuppression. Consequently, the net financial impact was estimated to be a budgetary cost saving.^48^

Other clinical contexts may also benefit from the assessment of treatment‐induced myelosuppression. Thoracic radiotherapy is often administered as consolidation therapy to patients with ES‐SCLC who respond to systemic chemotherapy.^49^, ^50^ In addition, the combination of immunotherapy plus radiotherapy is now being investigated in patients with ES‐SCLC, on the basis of improvements shown in the non‐SCLC setting.^51^ Like cytotoxic chemotherapy, radiotherapy damages rapidly proliferating bone marrow cells, which can lead to haematological toxicity.^52^ Indeed, in one retrospective analysis of patients with ES‐SCLC, there was significantly more leukopenia in patients receiving chemotherapy and radiotherapy than in those receiving chemotherapy alone (53% vs 34%; *P* = .033).^53^ Although the clinical effects of trilaciclib have not been investigated in patients receiving radiation therapy, it is feasible that the myeloprotective effects of trilaciclib when administered prior to chemotherapy may subsequently benefit patients treated in this setting by helping to protect HSPCs from damage before consolidation radiotherapy. The potential effects of trilaciclib in the context of radiotherapy therefore warrant further investigation.

Finally, although there is a theoretical concern that trilaciclib may antagonise the intended antitumor efficacy of chemotherapy in CDK4/6‐dependent tumours, trilaciclib had no impact on the efficacy of chemotherapy in the SCLC studies, or in preclinical models of CDK4/6‐dependent and ‐independent tumours.^25^, ^29^, ^30^, ^31^, ^32^, ^54^ In each of the three randomised trials included in this analysis, progression‐free survival and overall survival (OS) were similar between the trilaciclib and placebo arms.^29^, ^30^, ^31^ Moreover, in study G1T28‐05, median OS with trilaciclib prior to etoposide/carboplatin/atezolizumab (12.0 months) was similar to that observed with etoposide/carboplatin/atezolizumab in the pivotal IMPower133 study (12.3 months).^3^, ^29^

Strengths of this analysis include the predefined nature of the MAHE endpoint, and the consistent benefits observed with trilaciclib vs placebo prior to chemotherapy across multiple studies. The analysis was limited by the relatively small number of patients enrolled in these studies, as reflected by the fact that differences between trilaciclib and placebo were not consistently observed in individual studies for some of the MAHE components. Nonetheless, pooling of the datasets, which was supported statistically, allowed the effect of trilaciclib on these endpoints to be assessed with greater statistical accuracy. An additional limitation is that the impact of trilaciclib in combination with other commonly used second‐ or later‐line chemotherapy options for SCLC (aside from topotecan) was not evaluated. Overall, however, this analysis strengthens the conclusion that trilaciclib is well tolerated and acts as a myeloprotection agent, reducing CIM and its consequences across multiple haematopoietic cell lineages.

In conclusion, the robust improvements in the exploratory MAHE composite endpoint across the three studies and the pooled analysis further support the myeloprotective benefits of trilaciclib and its ability to improve the safety of chemotherapy regimens used to treat patients with ES‐SCLC. Using the MAHE endpoint to assess clinical and health economic outcomes may help to ensure optimal patient care.

## CONFLICT OF INTEREST

Dr Manuel Dómine Gómez has participated in lectures and advisory boards for AstraZeneca, Bristol Myers Squibb, Boehringer Ingelheim, MSD, Pfizer and Roche, and has received support for attending meetings and/or travel from AstraZeneca, Boehringer Ingelheim, Pfizer and Roche. Drs Tibor Csőszi, Iveta Kudaba, Krasimir Nikolov and Davorin Radosavljevic have no conflicts of interest to declare. Dr Jana Jaal has received research funding from G1 Therapeutics and AstraZeneca, has participated in advisory boards for AstraZeneca, Boehringer Ingelheim and MSD, and has received support for attending meetings and/or travel from AstraZeneca and MSD. Dr Jie Xiao was an employee and shareowner of G1 Therapeutics, Inc. at the time of study and manuscript preparation. Drs Janet K. Horton and Rajesh K. Malik are employees and shareowners of G1 Therapeutics, Inc. Dr Janakiraman Subramanian has consulted for AstraZeneca, Boehringer Ingelheim, Eli Lilly, G1 Therapeutics, Jazz Pharma, Novartis, Pfizer and Takeda, has participated as a speaker for AstraZeneca, Boehringer Ingelheim and Eli Lilly and has provided writing support for AstraZeneca, Boehringer Ingelheim and Novartis.

## Data Availability

The data that support the findings of this study are available from the corresponding author upon reasonable request.
